# Mode of Commuting to School and Its Association with Physical Activity and Sedentary Habits in Young Ecuadorian Students

**DOI:** 10.3390/ijerph15122704

**Published:** 2018-11-30

**Authors:** Yaira Barranco-Ruiz, Alfredo Xavier Guevara-Paz, Robinson Ramírez-Vélez, Palma Chillón, Emilio Villa-González

**Affiliations:** 1 Promoting Fitness and Health through Physical Activity Research Group (PROFITH), Department of Physical Education and Sports, Faculty of Sport Sciences, University of Granada, 18071 Granada, Spain; ybarranco@ugr.es or ybarranco@unach.edu.ec (Y.B.-R.); pchillon@ugr.es (P.C.); evilla@ugr.es or evilla@unach.edu.ec (E.V.-G.); 2 Escuela de Cultura Física, Universidad Nacional de Chimborazo, Riobamba 060110, Ecuador; alfredoguevarapaz@gmail.com; 3 Centro de Estudios Para la Medición de la Actividad Física CEMA, Escuela de Medicina y Ciencias de la Salud, Universidad del Rosario, Bogotá 111221, Colombia

**Keywords:** transport to school, mode of commuting, youth, young, physical activity

## Abstract

Active commuting to and from school (ACS) could help to increase daily physical activity levels in youth; however, this association remains unknown in Ecuadorian youth. Thus, the aims of this study were (1) to determine the patterns of commuting to and from school and (2) to analyze the associations between ACS, physical activity (PA), and sedentary habits in Ecuadorian youth. A total of 732 students (65.3% males), aged 10–18 years (children = 246, young adolescents = 310, older adolescents = 162) from the central region of Ecuador participated in this study. A self-report questionnaire, including the usual mode and frequency of commuting, distance from home to school (PACO-Questionnaire), and PA and sedentary habits (YAP-Questionnaire), was used. Most of the sample lived ≤2 km from school; however, they were mainly passive commuters (96%). The most common mode of commuting was by car (to school = 43.4%, from school = 31.6%; *p* < 0.001). Children presented significantly higher scores (0–4) in PA outside school and total PA compared with older adolescents (2.20 ± 0.97 vs. 1.97 ± 0.96; *p* = 0.013 and 2.30 ± 0.76 vs. 2.09 ± 0.74, *p* = 0.019, respectively), as well as the lowest scores in sedentary habits (1.51 ± 0.65, *p* < 0.001). PA at school and total PA were positively associated with ACS (OR 3.137; 95% CI, 1.918 to 5.131; *p* < 0.001, and OR 2.543; 95% CI, 1.428 to 4.527; *p* = 0.002, respectively). In conclusion, passive modes of transportation were the most frequently used to commute to and from school in young Ecuadorians. PA at school and total PA were positively associated with ACS. Thus, interventions at school setting could be an opportunity to improve PA levels and additionally ACS in youth from the central region of Ecuador.

## 1. Introduction

Physical inactivity has spread to many countries of the world in recent years [1]. As a consequence, a negative impact on the health of the population has been described, with an increased prevalence of several noncommunicable diseases (NCD), such as hypertension and cardiovascular diseases. Knowing that cardiovascular diseases progress through adolescence and early adulthood [2], the World Health Organization (WHO) [3] currently recommends that children and adolescents meet a target of at least 60 min of daily physical activity (PA) of moderate to vigorous intensity. Another recommendation based on WHO guideline MVPA, which was suggested by Colley et al. [4] was to perform at least 12.000 steps per day; however, until now, more than 80% of the world’s young population is insufficiently physically active (WHO, February 2018) [5].

There is evidence that active commuting is a source of physical activity that reduces the risk of cardiovascular disease [6], cancer, and chronic diseases in adults [7]. In youth, daily behaviors, such as active commuting to school (ACS), i.e., walking or cycling to and from school, might increase PA levels, further reducing the incidence of metabolic syndrome [8]. However, ACS behavior depends on different sociocultural and/or environmental factors, such as parents’ perception of safety [9], the distance from home to school [10], or even the weather [11].

The prevalence of ACS varies across European countries, such as in Swedish children (63%) [12], Spanish adolescents (50%) [13], Portuguese children (21%) and adolescents (45%) [14], or German adolescents (≈21%) [15]. A similar variety occurs in South American countries, with Chilean children (11.0%) and adolescents (25%) [16], Brazilian children (41%) [17], and Colombian children and adolescents (22%) [8]. However, more studies about ACS are needed in the Latin American population to provide a broader overview; furthermore, there are no ACS data on youth from Ecuador.

The latest Ecuadorian government report [18] found that Ecuadorian children between 10 and 18 years of age presented a highly sedentary lifestyle, with 20.2% of them being sedentary for 2–4 h daily and 5.4%, for >4 h daily. This report pointed out the need to promote PA interventions in Ecuador. Therefore, we consider ACS a potential strategy to improve PA levels and vice versa, especially applying intervention programs focused on ACS, such as the Safe Routes to School, but the curriculum recommends the use of 5 strategies (encouragement, education, enforcement, environment, and evaluation) in order to promote active transport to school interventions [19]. To our knowledge, there are no previous cross-sectional or intervention studies reporting data about ACS patterns and their association with PA levels and sedentary habits in the young Ecuadorian population. Therefore, the aims of the current study were (1) to determine the patterns of commuting to and from school and (2) to analyze the association between ACS, PA, and sedentary habits in Ecuadorian youth.

## 2. Materials and Methods

### 2.1. Subject Population

The data were collected between April 2014 and May 2015, from the first phase of the “Cycle and Walk to School” Ecuador research project (original name in Spanish “Proyecto PACO: Pedalea y Anda al Colegio”). This present study examined ACS patterns in Ecuadorian children and adolescents. The Medical Ethics Committee of the National University of Chimborazo (Riobamba, Ecuador) approved the study design, study protocols, and informed consent procedure (ID: 46-CI-2015-07-02).

Three schools from Riobamba (Central region of Ecuador) selected via convenience agreed to participate in this study. A total of 732 students consented to participate; specifically, 246 children (aged from 10 to 12 years), 310 young adolescents (aged from 13 to 15 years), and 162 older adolescents (aged from 16 to 18 years) [20]. An additional 14 participants did not report their age. The mean age of the total sample was 13.7 ± 2.1 years, and male was the predominant gender (65.3%), especially in the older adolescent group (73.5%). The study sample was homogeneous regarding socio-economic status (SES), presenting a low to middle SES (1 to 3 defined by the Ecuadorian government).

### 2.2. Questionnaire

The “PACO” (Pedalea y Anda al Colegio) questionnaire, which was created by the “PACO” project at the University of Granada (Granada, Spain), was used in this study. This questionnaire (http://profith.ugr.es/paco) included previously validated questions about (1) the mode of commuting to and from school [21] and (2) the distance from home to school [22], as well as (3) the YAP (youth activity profile) questionnaire, which is a previously validated, self-administered 7-day reminder survey for the young population focusing on collecting information about PA and sedentary habits in the previous week [23].

The research team adapted the questionnaire to the Ecuadorian cultural context following this process: (a) Two different researchers adapted the questions to the Ecuadorian context; (b) a third researcher combined the adaptations; and (c) finally, the questionnaire was presented to 10 young people to obtain their opinions. This process assured comprehension of the questionnaire, and some expressions were changed from the Spanish version (e.g., “*escuela*” replaced the original term of “*centro educativo*”). The participants completed this questionnaire (approximately 20–30 min) with guidance from the investigators (ratio 1:15) and their teacher in the physical education class.

### 2.3. Modes of Commuting to School

The usual mode of commuting to and from school was analyzed using the following question: How do you usually travel to and from school? Modes of commuting were categorized as active, i.e., walking and cycling, or passive, i.e., by car, motorcycle, school bus, public bus and subway, train or tram. Since there is no subway, train or tram in Riobamba, these categories were omitted, as was “other”, since the participants did not describe any other modes of commuting.

In addition, participants were categorized as active or passive commuters to and from school using as criteria the frequency of the weekly commute to and from the school for each mode of commuting analyzed by the following questions: How do you travel to and from school each day? Participants were classified as active commuters if they made at least 2 full round trips to school (4 out of 10 possible trips/week) using active modes of transport and were classified as passive commuters if they did not comply with the minimum number of weekly trips (4 out of 10 possible trips/week) using active modes of transport [21].

### 2.4. Distance to School

The distance to school was analyzed using the question: *How far away do you live from school?* Responses were categorized as “≤2 km”, “2–5 km” or >5 km” [24]. The distance was also calculated objectively through Google Maps^TM^, selecting the shortest on-foot network path between each student’s home address and the school, measured in meters [25].

### 2.5. Youth Activity Profile

To establish PA and sedentary profiles, the YAP questionnaire was used [23]. This questionnaire was based on the PA questionnaires for children (PAQ-C) [26] and adolescents (PAQ-A) [27]. The YAP includes 15 questions covering three categories of PA and sedentary habits in youth: (1) PA at school; (2) PA outside of school; and (3) sedentary habits. Each category presented five questions, and responses were scored from 0 to 4. To assess the total score for each section, the average of the scores (0 to 4) obtained for each section was calculated, whereas to obtain the score for the total PA profile, the sum of mean scores for the PA at school and PA outside of school sections was calculated. Items within the school section included participation in moderate to vigorous physical activity (MVPA) during 5 specific windows along the day (transportation to and from school, as well as activity during physical education, lunch, and recess). Items in the out-of-school section included activities before school, right after school, during the evening, and on each weekend day (Saturday and Sunday). Sedentary items included time spent watching TV, playing videogames, using the computer, using a cell phone, and also an overall sedentary time item.

### 2.6. Statistical Analysis

Data are presented as mean and standard deviation of the mean for quantitative variables and as frequencies and percentages for qualitative variables. The distribution of the study variables was analyzed by employing a Kolmogorov–Smirnov test. The McNemar test was carried out to compare the percentages of each mode of transport in the trip from home to school and in the trip from school to home, in the total sample of participants and by age group. The chi-square test was used to analyze differences between the percentages of students who used each type of commuting mode (active or passive) according to age group. The Kruskal–Wallis test was performed to analyze the differences in the mean scores obtained for all the YAP questionnaire categories by age group. The scores for PA at school and total PA were also calculated independently of the answers to questions about commuting. To analyze the association between the different categories of the YAP questionnaire (independent variable) and the probability of being an active commuter to and from school, i.e., at least 4 out of 10 possible trips/week using an active mode of transport (dependent variable), binary logistic regressions were performed according to the different age groups, using age, gender, and distance as covariates. Significance was established at *p* < 0.05. All analyses were carried out using the SPSS^®^ 22 statistical package (IBM, Armonk, New York, USA).

## 3. Results

Most of the participants, regardless of age group, lived less than 2 km from school (38.4%), Table 1.

**Figure 1:**
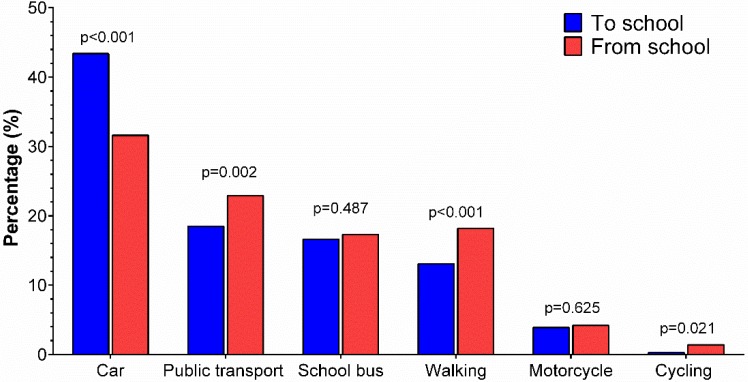
Mode of commuting to and from school for the whole sample.

The usual modes of commuting to and from school in the overall sample are presented in Figure 1. The main modes of commuting used for this sample were passive, such as by car, public transport or school bus, whereas active modes (i.e., walking and cycling) were less often used. Significant differences between the percentage in the trip to school and from school were observed in the following modes of commuting: Car (*p* < 0.001), public transport (*p* = 0.002), walking (*p* < 0.001), and cycling (*p* = 0.021).

**Figure 2:**
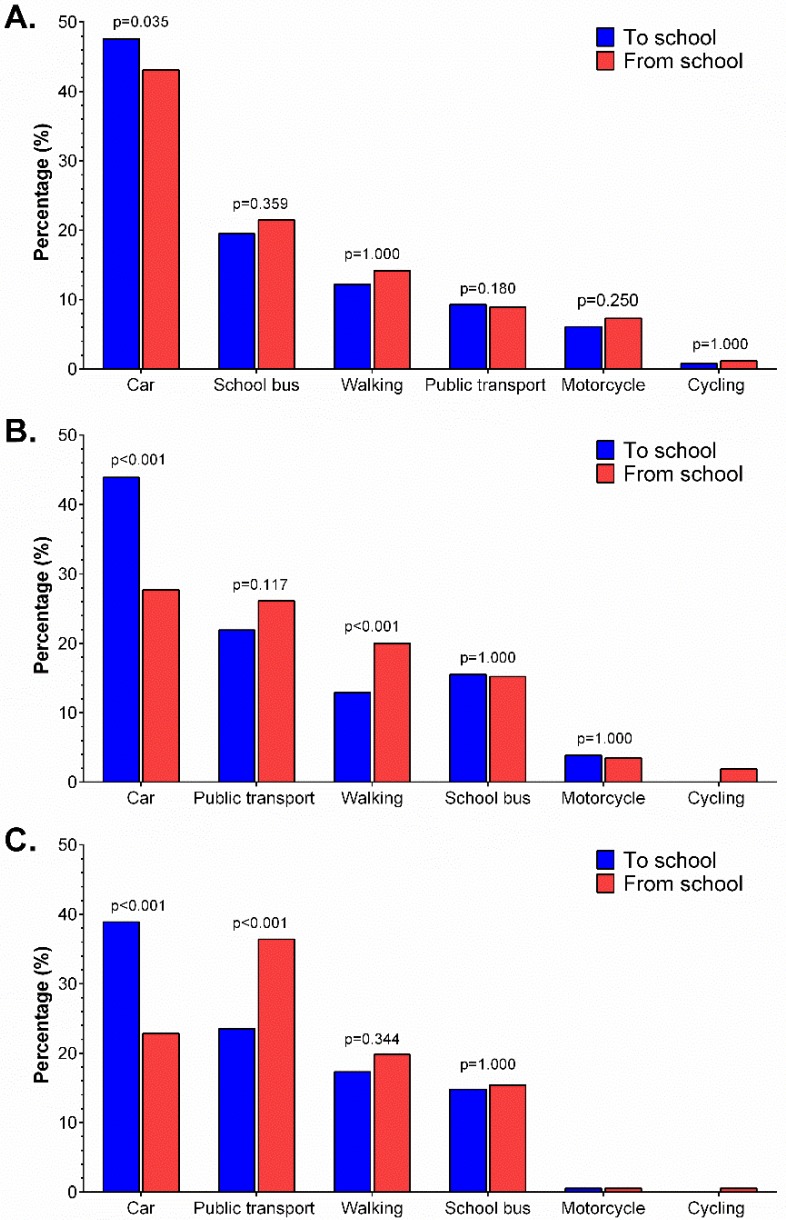
Modes of commuting to and from school according to age groups. A. Children, B. Young Adolescents and C. Older Adolescents.

The modes of commuting to and from school by age group are presented in Figure 2. In descending order, the usual mode of commuting to and from school for young adolescents and older adolescents was by car, public transport, walking, school bus, motorcycle, and bicycle. However, in the case of children, scholar bus reached the second position instead of public transport. Significant differences between the trip to and from school were found for the use of the car in all age groups (children: *p* = 0.035, and young adolescents and older adolescents: *p* < 0.001, both respectively), where the use of the car was significantly lower for the trip from school to home than for the trip from home to school. Additionally, young adolescents showed a significant increase for walking mode of commuting from school to home (*p* < 0.001), and older adolescents significantly increased the use of the public transport for going back home (*p* < 0.001). In general, the usual modes of commuting to and from school used by the study participants were passive (i.e., car, school bus, and public transport), independently of the age group, while active commuting modes were the least used. The use of the car as the mode of commuting during the trip back from school to home significantly decreased compared to the trip to school for all age groups.

The prevalence by type of commuting and the average scores for the different categories of the YAP-Q (i.e., PA at school, PA outside of school, sedentary habits, and total PA) are presented in Table 2 for the overall sample and categorized by age groups.

No significant differences were found between age groups in the variable “type of commuting” (passive/active) (*p* = 0.255). The highest average score on the YAP questionnaire was presented in the PA at school category for the entire sample (2.27 ± 0.82) and for all age groups. When the average scores of the different categories of the YAP questionnaire were compared according to age group, significant differences were observed in general for PA outside of school (*p* = 0.043) and sedentary habits (*p* < 0.001), as well as YAP categories that did not involve questions about commuting (*p* < 0.001).

After post hoc analysis for the comparisons of YAP-Q categories between each age groups, children attained a significantly higher score in PA outside of school and total PA than older adolescents (2.20 ± 0.97 vs. 1.97 ± 0.96; *p* = 0.013 and 2.30 ± 0.76 vs. 2.09 ± 0.74, *p* = 0.019, respectively) and a significantly lower score in sedentary habits compared with young (1.51 ± 0.80 vs. 1.72 ± 0.72; *p* < 0.001) and older adolescents (1.51 ± 0.80 vs. 1.78 ± 0.66; *p* < 0.001). Regarding the YAP categories without including the commuting questions, children presented higher scores in PA at school than young adolescents (*p* = 0.042) and older adolescents presented the lowest score in PA at school and total PA compared to all age groups (children: *p* < 0.001, young adolescents: *p* = 0.003).

**Figure 3:**
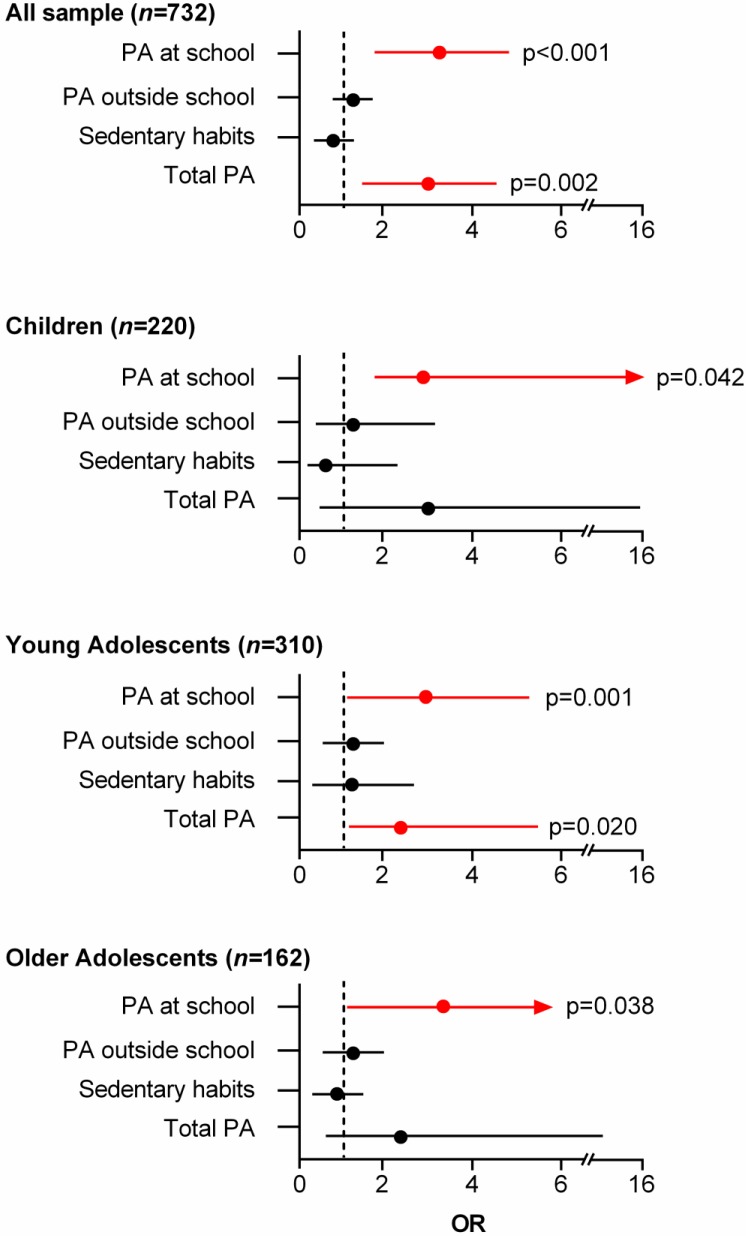
Association between be active commuting to and from school and YAP-Q categories for the whole sample and by age groups. Red arrows indicate significant association.

Figure 3 shows the associations between the different categories of the YAP questionnaire and ACS for the whole sample and categorized by age groups. For the whole sample, PA at school and total PA were positively associated with ACS (OR 3.13; 95% CI, 1.91 to 5.13; *p* < 0.001, and OR 2.54; 95% CI, 1.42 to 4.52; *p* = 0.002, respectively). There was also a significant association between a higher score in PA at school and ACS when categorized by age group (children: OR 3.26; 95% CI, 1.04 to 10.19, *p* = 0.042, young adolescents: OR 3.00, 95% CI, 1.58 to 5.70, *p* = 0.001 and older adolescents: OR 3.34; 95% CI, 1.07 to 10.42, *p* = 0.038). All participants with a score between 2.20 and 2.34 in PA at school presented a high probability (children: 76.5%, young adolescents: 75%, and older adolescents: 76.9%) of performing ACS (4 out of 10 possible trips/week). PA outside the school and sedentary habits were not associated with ACS for any of the age groups. Notwithstanding this, total PA was positively associated with ACS in young adolescents (OR 2.44; 95% CI, 1.15 to 5.15, *p* = 0.020); with a score of 2.20 in total PA, they reached a 70% probability of performing ACS (4 out of 10 possible trips per week).

## 4. Discussion

The main findings of this study indicate that total PA and PA at school were positively associated with ACS in youth from the Andean central region of Ecuador. Most of the participants (96.6%) were passive commuters to and from school (less than 4 ACS trips/week), using mainly cars or public transport, despite most of them living ≤2 km from school.

In the current study, the most common mode of commuting used by students in all age groups was by car (43.4% and 31.6% to and from school, respectively), whereas active modes such as walking and cycling were less used (13.1%, 18.2% to and from, respectively, for walking and 0.3% and 1.4% to and from, respectively, for cycling). Similarly, results from other Latin American countries showed that the main mode of commuting to and from school was by car, such as in Chilean children and adolescents, with frequencies of 58.05% and 37.45%, respectively [16]. Conversely, 23% of Colombian young people (13.2 years old) reported commuting by cycling [8] and 62.5% of Brazilian students actively commuted to and from school [17]. To explain the differences in the rates of active commuting to and from school between studies, differences in the cultural, social, demographic and urban environments must be considered.

Regarding the distance from home to school, most participants lived ≤2 km from school, independently of age group. This fact, together with the majority of the sample being passive commuters, is a cause for concern, since previous studies showed that the threshold distances for active commuting are ≈1.5 km for walking and ≈3.0 km for cycling to and from school [10]. In agreement with our results, another study found that 44% of Chilean youth lived 2 km or less from school [16] and the rate of commuting on foot was similar (11.0% and 24.8% of children and adolescents, respectively) to ours. Previous studies from different regions of Ecuador have evidenced several perceived barriers to ACS which could influence its low rate, such as crime in urban areas [28], heavy traffic congestion [29], traffic volume and traffic speed [30], or even pedestrian crashes and car accidents [31].

Passive modes of commuting were the main modes used in the present study by all age groups, with significant differences between modes of commuting both to and from school. Along these lines, active commuting home from school exhibited a higher prevalence in the adolescent groups compared with the children group (young adolescents = 20% and older adolescents = 19.8% vs. children = 14.2%), in agreement with previous studies, which indicated that adolescents have more independent mobility than children, especially on the way home from school [32]. In the Spanish study, as perhaps in the present study, older children were more likely to commute to school unaccompanied, were more likely to commute actively, and had better safety perceptions than younger children. Finally, in the present study, all age groups presented a decrease in the use of the car for the trip back to home, where adolescents groups presented a greater decrease (16%) compared to children (4.5%). In line with this, other modes of commuting increased in the trip to home. For example, in the children group, the use of school bus and walk from school to home slightly increased (not significant differences), while in the young adolescents group, walk to home increased significantly, and public transport increased in the case of the older adolescents group (*p* < 0.001). Correspondingly, in a similar study conducted in Chile [16], adolescents reported using the public bus as the second most common mode of commuting for going back from school to home compared to children. We cannot determine the justification of these findings with accuracy, but we can hypothesize that factors such as a lack of time, independence, or family decisions may explain our results. In addition, that fact could be linked with the greater use of public transport in the transition from adolescence to adulthood [33]. Notwithstanding this, it is important to differentiate between public bus and school bus since the public bus has been cited as a way to help individuals to incorporate regular PA into their day, since people have to walk to the public transport stops [24,34,35], whereas school bus stops are usually located near the school. These differences between modes of commuting to and from the school should be studied in depth in order to include successful strategies to reconvert this behavior into an active habit.

According to the latest data provided by the Government of Ecuador [18], a large percentage of Ecuadorian children and adolescents are sedentary. Regarding PA levels, 34% of Ecuadorian adolescents were inactive, while 38.1% were irregularly active; therefore, fewer than 3 in 10 young people were active [18]. In our study, unlike in the Ecuadorian report, total PA decreased slightly with age (i.e., from childhood to older adolescence). Moreover, the participants in this study attained the highest scores for PA at school. Accordingly, in a previous Portuguese study [36], a higher proportion of time spent on MVPA was found in the transport to and from school domain (45.5%) and at school (30.5%), compared to the leisure time (21.3%) or home (2.7%) domains.

Concerning the association between total PA and ACS, our study demonstrated a positive association. Similarly, the HELENA study displayed a positive association between ACS and PA levels in European adolescents [13]. Additionally, in a study conducted with American children, ACS was associated with high levels of PA, in particular PA of moderate-to-vigorous intensity [37]. In a previous systematic review [38], children using ACS accumulated more daily PA than passive commuters in 40 studies (81.6%). Twelve of the fourteen studies that used self-report instruments showed that active commuters were more active across the full day (85.7%). Moreover, the two studies that used accelerometers in combination with a questionnaire found that walkers and cyclists evaluated by both instruments were significantly more active. In the same direction of our results, a Portuguese study showed a positive association between different PA domains and an active transportation to and from school [39]. Thus, this fact points to a bidirectional effect between ACS and PA; therefore, it would be advisable to analyze this association in both directions.

Our findings indicated that total PA was positively associated with ACS for the whole sample, and in particular, with PA at school when the sample was analyzed by age group (children, *p* = 0.042; adolescents, *p* = 0.001; and older adolescents, *p* = 0.038). This association could be expected, since ACS is part of the PA at the school category within the YAP-Q; however, the analyses were repeated without commuting questions, and the results remained constant for the whole sample (ACS and PA at school without commuting questions: OR 1.89; 95% CI, 1.19 to 2.99; *p* = 0.006, and ACS and total PA without commuting questions: OR 1.89; 95% CI, 1.08 to 3.29; *p* = 0.024). Previous studies have analyzed the association between different forms of PA at school setting and ACS. Walking to school was associated with a higher number of school-day steps in older children [40] and school sport participation was positively associated to ACS in Portuguese adolescents [39]. However, none of the studies have used the YAP-Q as instrument to analyze PA and specifically PA at school including several domains, i.e., PA during transportation to/from school, physical education classes, lunch, and recess. Our hypothesis is that those daily active behaviors that occur close to the transport to and from school can positively influence ACS. However, there is no previous evidence to support such a hypothesis. Thus, future studies should analyze the role that accumulated PA at school setting plays on other PA domains, such as transportation to and from school. Nonetheless, few studies have analyzed whether the association is more robust with PA at school or outside of school. A previous study reported that children who walked to school accumulated 30 additional minutes of MVPA outside of school [41]; however, in our study, ACS was only associated with PA at school, not outside of school. Finally, in the current study, children presented higher scores regarding PA outside of school compared to those of older adolescents, and the lowest scores for sedentary habits. Accordingly, several previous studies observed lower PA levels and higher levels of sedentary habits with increasing age [42,43,44], although many studies have found inconsistencies when comparisons were carried out between age groups [38]. Consequently, comparison with previous studies should be done with caution, since some studies used participant reports, whereas others used proxy reports from parents. Furthermore, findings were reported using different metrics (i.e., daily PA, PA index, proportion of participants meeting PA guidelines, PA profiles, etc.). In general, the literature results point to a positive association between performing ACS and PA, although objective measures or a combination (i.e., accelerometry and a questionnaire) are required for future studies. Thus, strategies to promote ACS must be especially considered in Latin American middle-income countries such as Ecuador, where there are low PA levels in the young.

We found several limitations in this study, such as the nonrandom, purposive sampling method, which may have produced a sample not representative of the population at large. In addition, data were collected using a self-report questionnaire; therefore, these results must be supported by studies employing objective measurements of PA levels, such as accelerometry or GPS, to evaluate the real distance of the route from home to school, despite the use of a validated instrument in this study (i.e., Google Maps^TM^). As a strength, to our knowledge, there are no studies that describe the patterns of commuting to and from school, PA, and their association in Ecuadorian students, despite the high rates of sedentary lifestyle and obesity that have been established in this country.

## 5. Conclusions

Young people from the Andean central region of Ecuador are mainly passive commuters to and from school, although most participants live 2 km or less from school. Moreover, PA at school and total PA are positively associated with ACS in Ecuadorian youth. Multidisciplinary partnerships between urban planners, landscape architects, public health experts, and schools should be fortified to construct strategies focusing on increasing ACS. Finally, educational programs at school and promotional campaigns are essential to develop positive attitudes, social support, and increase awareness of the benefits of walking.

## Figures and Tables

**Table 1:** Sociodemographic data of the participants.

Characteristics	Total Sample (*n* = 732)	Children (*n* = 246)	Young Adolescents (*n* = 310)	Older Adolescents (*n* = 162)
**Gender**				
Male	477 (65.3)	138 (56.1)	211 (68.3)	119 (73.5)
Female	254 (34.7)	108 (43.9)	98 (31.7)	43 (26.5)
**Age (years)** *	13.7 (2.1)	11.2 (0.7)	14.1 (0.7)	16.4 (0.35)
**Distance (km)** *	3.2 (3.0)	2.8 (2.7)	3.1 (3.1)	3.8 (3.3)
≤2 km	267 (38.4)	93 (38.8)	115 (38.6)	59 (37.6)
2–5 km	226 (32.4)	82 (34.2)	92 (29.7)	52 (33.1)
>5 km	202 (29.2)	65 (27.1)	91 (28.4)	46 (29.3)

Data are presented as frequency and percentage n (%) of the overall sample and according to age groups. * Mean and standard deviation (SD).

**Table 2:** Type of commuting to and from school and youth activity profile.

Characteristics	Total (*n* = 735)	Children (*n* = 246)	Young Adolescents (*n* = 310)	Older Adolescents (*n* = 162)	*p* value	
Type of commuting *	Passive (*n* = 707)	Passive (*n* = 240)	Passive (*n* = 294)	Passive (*n* = 156)	0.255	
	Active (*n* = 28)	Active (*n* = 6)	Active (*n* = 16)	Active (*n* = 6)		
**YAP-Q categories ^#^**					Kruskal-Wallis test	Mann-Whitney test (Age groups comparisons)
*PA at school*	2.27 (0.82)	2.34 (0.80)	2.26 (0.81)	2.20 (0.86)	0.210	0.189
*PA outside the school*	2.11 (0.94)	2.20 (0.97) ^b^	2.14 (0.92)	1.97 (0.96)	0.043	0.013
*Sedentary habits*	1.65 (0.73)	1.51 (0.80) ^a,b^	1.72 (0.72)	1.78 (0.66)	<0.001	<0.001
*Total PA*	2.20 (0.74)	2.30 (0.76) ^b^	2.20 (0.73)	2.09 (0.74)	0.059	0.019
PA at school **	2.96 (0.94)	3.13 (0.95) ^a,b^	2.95 (0.93) ^c^	2.70 (0.90)	<0.001	<0.001
Total PA **	2.54 (0.77)	2.67 (0.79) ^b^	2.55 (0.74) ^c^	2.33 (0.77)	<0.001	0.003

^#^ Mean and standard deviation (SD). PA = Physical activity; YAP-Q = Youth Activity Profile questionnaire; * Participants were categorized as active when they used to commute to and from school (4 out of 10 possible trips/week) using active modes of commuting; ** YAP-Q categories without commuting questions; *p* = significative differences according to age groups (Chi-square test was used for comparing type of commuter; Kruskal–Wallis test for the YAP-Q categories); Mann–Whitney was used to analyze the following comparisons: ^a^ Children vs. young adolescents; ^b^ children vs. older adolescents; ^c^ young adolescents vs. older adolescents.
